# Neurocognitive correlates of the effects of yoga meditation practice on emotion and cognition: a pilot study

**DOI:** 10.3389/fnint.2012.00048

**Published:** 2012-07-26

**Authors:** Brett E. Froeliger, Eric L. Garland, Leslie A. Modlin, F. Joseph McClernon

**Affiliations:** ^1^Department of Psychiatry and Behavioral Sciences, Duke University Medical CenterDurham, NC, USA; ^2^Brain Imaging and Analysis Center, Duke University Medical CenterDurham, NC, USA; ^3^College of Social Work, Florida State UniversityTallahassee, FL, USA; ^4^Trinity Institute for the Addictions, Florida State UniversityTallahassee, FL, USA

**Keywords:** mindfulness, fMRI, emotion-cognition

## Abstract

Mindfulness meditation involves attending to emotions without cognitive fixation of emotional experience. Over time, this practice is held to promote alterations in trait affectivity and attentional control with resultant effects on well-being and cognition. However, relatively little is known regarding the neural substrates of meditation effects on emotion and cognition. The present study investigated the neurocognitive correlates of emotion interference on cognition in Yoga practitioners and a matched control group (CG) underwent fMRI while performing an event-related affective Stroop task. The task includes image viewing trials and Stroop trials bracketed by neutral or negative emotional distractors. During image viewing trials, Yoga practitioners exhibited less reactivity in right dorsolateral prefrontal cortex (dlPFC) to negative as compared to neutral images; whereas the CG had the opposite pattern. A main effect of valence (negative > neutral) was observed in limbic regions (e.g., amygdala), of which the magnitude was inversely related to dlPFC activation. Exploratory analyses revealed that the magnitude of amygdala activation predicted decreased self-reported positive affect in the CG, but not among Yoga practitioners. During Stroop trials, Yoga practitioners had greater activation in ventrolateral prefrontal cortex (vlPFC) during Stroop trials when negative, compared to neutral, emotional distractor were presented; the CG exhibited the opposite pattern. Taken together, these data suggest that though Yoga practitioners exhibit limbic reactivity to negative emotional stimuli, such reactivity does not have downstream effects on later mood state. This uncoupling of viewing negative emotional images and affect among Yoga practitioners may be occasioned by their selective implementation of frontal executive-dependent strategies to reduce emotional interference during competing cognitive demands and not during emotional processing *per se*.

## Introduction

Hatha Yoga is a 600 year old practice that integrates physical poses (i.e., asana), meditation, breath work (i.e., pranayama), study of tantric philosophy and community outreach. The practice of yoga is shown to improve cognition in healthy (Manjunath and Telles, 2004) and clinical populations including multiple sclerosis (Velikonja et al., 2010) and major depressive disorder patients (Sharma et al., 2006). Yoga has also proven effective for improving emotional function in healthy (Hartfiel et al., 2011) and in clinical populations including reducing negative affect (Vadiraja et al., 2009), anxiety (Vadiraja et al., 2009; Streeter et al., 2010), depression (Banerjee et al., 2007), and improving emotional well-being (Moadel et al., 2007). These observed beneficial effects on cognitive and emotional health are thought to result in part from increased mindfulness arising from various yoga practices (Salmon et al., 2009).

Hatha yoga involves mindfulness *practice*, that is, repeated placement of attention onto an object while alternately acknowledging and letting go of distracting thoughts and emotions. Within the Hatha yoga tradition, mindfulness practice occurs both during the physical postures as well as during formal mindfulness meditation on one's breathing, where the object of mindfulness practice might include proprioceptive or interoceptive sensations stemming from physical posture or respiration, respectively. In turn, mindful yoga practices may generate the *state* of mindfulness, which, when evoked recurrently through repeated practice, may accrue into *trait* or *dispositional* mindfulness (Chambers et al., 2009; Garland et al., 2010). The state of mindfulness is characterized by a nonjudgmental and metacognitive monitoring of moment-by-moment cognition, emotion, perception, and sensation without fixation on thoughts of past and future (Kabat-Zinn, 1982; Garland, 2007; Lutz et al., 2008). Correspondingly, trait mindfulness is characterized as the tendency to adopt a nonjudgmental awareness of one's thoughts, emotions, experiences, and actions in everyday life (Baer et al., 2006). Trait mindfulness can be promoted by recurrent mindfulness practice. For example, individuals participating in an eight-week Mindfulness-Based Stress Reduction course evidenced increases in trait mindfulness which mediate the effects of training on clinical outcomes (Carmody and Baer, 2008; Greeson et al., 2011). Moreover, participants in a yoga intervention exhibited significant increases in trait mindfulness after eight weeks of training (Shelov et al., 2009).

Insofar as the practice of mindfulness generates state mindfulness via intentional attending to emotions without cognitive fixation or elaborative processing of emotional experience, this practice may produce positive effects on emotion-cognition interactions. For example, mindfulness practice has been shown to result in improved ability to regulate negative emotions (Chiesa and Serretti, 2010), enhanced attentional orienting (Jha et al., 2007; Lutz et al., 2008), and increased cognitive flexibility (Wenk-Sormaz, 2005; Zeidan et al., 2011). In light of these short-term benefits, long-term mindfulness practice has the potential to promote durable alterations in trait affectivity and attentional control with resultant effects on well-being and cognitive function. These salutary, trait-level effects may be observed in identified positive associations between trait mindfulness and enhanced affect regulation (Chambers et al., 2009), attentional control (Moore and Malinowski, 2009), and autonomic recovery from emotional provocations (Garland, 2011). Plausibly, such lasting functional improvements may derive from mindfulness-induced neuroplasticity in brain regions instantiate cognition and emotion (Hölzel et al., 2011).

### Neurocognitive model of emotion-cognition interactions

Outside of the context of yoga, meditation, or mindfulness, a prevailing neurobiological model posits that affective and cognitive processes are coordinated via an interaction between a dorsofrontal executive network and a ventral-affective circuit (Mayberg, 1997; Drevets and Raichle, 1998). Task-relevant targets activate the dorsolateral prefrontal cortex (dlPFC), whereas emotional distractors activate the amygdala (Yamasaki et al., 2002). Exerting cognitive control over emotional processes leads to increased activation in the dlPFC, with corresponding reciprocal deactivation in the amygdala (Ochsner et al., 2002; Ochsner and Gross, 2008).

A nascent database has emerged on the neurocognitive correlates of yoga and meditation practice. Neuroimaging research has demonstrated differences in task-related brain function between experienced meditation practitioners and meditation naïve controls. For example, fMRI analyses indicate that meditation practitioners exhibit greater meditation-related neural activation in brain regions involved in attentional control (e.g., prefrontal cortex), conflict resolution (e.g., dorsal anterior cingulate cortex) and emotional processing (e.g., medial/orbitofrontal cortices) (Hölzel et al., 2007; Baron Short et al., 2010). Moreover, compared to non-meditators, mindfulness practitioners evidence attenuated electrophysiological activation in frontal scalp regions to negative emotional stimuli during passive picture viewing (Sobolewski et al., 2011), and yoga meditation practitioners (YMP) exhibit sustained reductions in the late positive brain potential during cognitive reappraisal of negative emotional stimuli (Gootjes et al., 2011) and reduced power in high-frequency EEG spectrum during negative emotional information processing (Aftanas and Golosheykin, 2005). In addition, on an auditory oddball task, mindfulness meditation reduced N1, P2, and P3a amplitude to distractor stimuli (Cahn and Polich, 2006). Taken together, these findings suggest that meditation practitioners evidence significantly different neural responses in cognitive and affective brain circuitry than non-meditators which may mediate the identified salutary effects of mindfulness practice on attentional and emotional processes. Though these data provide important neural clues for the effects of Yoga meditation (YM) practice on emotional and cognitive processes, the precise neural mechanisms underlying the effects of YM on emotion-cognition interactions (e.g., emotional information processing; emotional interference on cognition) remain largely unknown.

### Current study

The present study investigated the neurocognitive correlates of emotional interference on a cognitively demanding task within a sample of meditation practitioners and matched controls. In the present study, we sought to investigate the effects of YM on emotion-cognition interactions. YMP and a matched control group (CG) of yoga and meditation naïve subjects underwent fMRI scanning while performing an Affective Stroop Task (Blair et al., 2007; Vythilingam et al., 2007; Hasler et al., 2009; Mueller-Pfeiffer et al., 2010; Froeliger et al., 2011, 2012), a modified version of the Number Stroop task (Pansky and Algom, 2002). The Affective Stoop task was designed to evaluate emotional information processing and its effects on cognitive conflict resolution. We hypothesized that YMP, as compared to the CG, would exhibit less brain activation [i.e., Blood-oxygenation-level-dependent (BOLD) response] during negative emotional information processing and greater brain activation during Stroop trials in the executive control system (e.g., PFC).

## Materials and methods

### Participants

Fourteen [7 Hatha YMP, 7 Hatha yoga and meditation-naïve control (CG)] participants between the ages of 18 and 55 years were enrolled. MP participants reported engaging in mindfulness meditation on average 7 days per week [0] over the course of the previous 5.7 yrs [3.8]. In addition, participants in the YMP group were also involved in an active and ongoing hatha yoga practice (>45-min a day, three-four times per week, >3 years). The matched CG reported no current or past dedicated meditation or yoga practice. In addition, all participants were right-handed, free of any psychiatric condition or any major medical condition that would make participation unsafe or uncomfortable. Additional exclusionary criteria included current alcohol or drug abuse, use of tobacco or nicotine products and positive urine drug screen. Female participants were required to have a negative urine pregnancy test at screening and within 12 h prior to the fMRI scan. The protocol was approved by the institutional review board at Duke University Medical Center, and all participants provided written informed consent before participating in study-related activities.

### Procedures

After screening, eligible participants completed one training session during which they practiced the experimental task and were placed in a mock scanner in order to habituate to the scanning environment. Following training, participants completed one fMRI session.

### Assessment of trait and state affect

Baseline measures included assessment of depressive symptoms with the Center for Epidemiological Studies-Depression (CES-D) scale (Radloff, 1977) and anxiety symptoms with the Beck Anxiety Inventory (BAI). State-dependent mood was measured using the 20-item positive and negative affect schedule (PANAS) (Watson et al., 1988). This measure results in two orthogonal scales—Positive Affect (attentive, proud) and Negative Affect (distressed, angry).

### Affective stroop task

**Figure 1:**
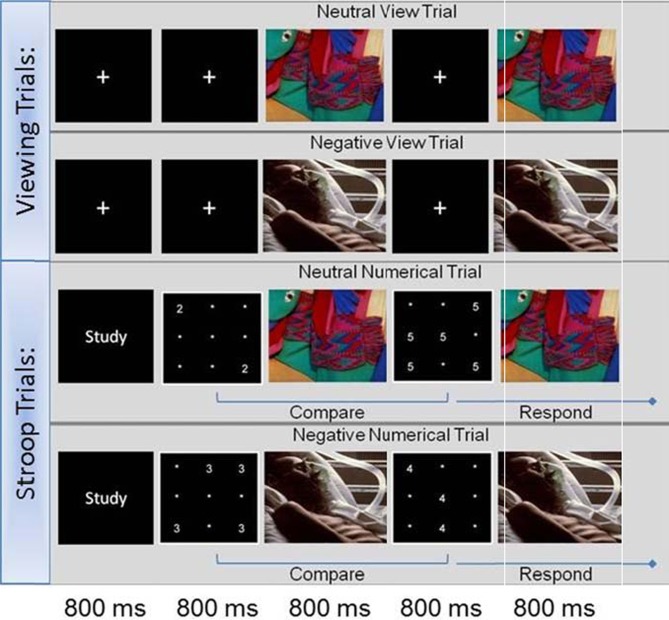
**Affective Stroop task**.

The Affective Stroop Task used in the present study was similar to that used in other studies evaluating emotion-cognition interactions (Blair et al., 2007; Vythilingam et al., 2007; Hasler et al., 2009; Mueller-Pfeiffer et al., 2010; Froeliger et al., 2011). During each imaging session, participants performed two runs of the task (Figure 1). Stimuli consisted of number grids and distractor images. The number grids consisted of numerals (1's through 6's) randomly presented within a 9-point grid-field. Distractor stimuli were negative and neutral valence images selected from the International Affective Picture Series (IAPS) (Lang et al., 1997) on the basis of 9-point arousal (1-lowest, 9-highest) and valence (1-negative, 5-neutral, 9-positive) scales. Valence and arousal ratings for chosen images did not overlap across categories (Negative, Valence <3, Arousal >6; Neutral, Valence 4 to 6, Arousal <3) and were matched on mean luminance, chromatic features, and scene complexity. Within the aST, two primary types of trials are randomly presented, Stroop trials that contain numerical grids and distractor images, and viewing trials—requiring the participant to only view images (Figure 1). The Stroop trials began with a fixation cross, followed by a number grid, a negative or neutral distractor image, a unique number grid, and concluded with the re-presentation of the distractor image each for 800 ms. Participants were instructed to report by button press which number grid presented (1st or 2nd) contained greater numerosity (the quantity of numbers presented) as quickly and accurately as possible. Stroop trials were further broken down into two subcategories: congruent and incongruent trials. During congruent trials, the number grids presented numerals with a face value congruent with the numerosity (e.g., two 2's; five 5's). During the incongruent trials, the face value of the numerals did not match the numerosity (e.g., four 3's; three 4's). Response accuracy and reaction times (RT's) were recorded for each trial. During the viewing trials the numerical grids were replaced with a crosshair and no responses were recorded. Finally, numerical trials began with a brief (800 ms) instruction “Study”; whereas emotional information processing trials began with a brief (800 ms) instruction “View”. During each 8 1/2 min run, six event types (negative congruent, negative incongruent, neutral congruent, neutral incongruent, negative view, and neutral view) were each randomly presented equally (15 events), resulting in a total of 30 events per type during each scanning session.

### Analysis of behavioral data

Analyses of the effects of group on overall task response RT and accuracy during the Stroop trials were conducted using a 2 (Group: YMP, control) × 2 (Task Condition: congruent, incongruent) × 2 (Distractor Valence: negative, neutral) ANOVA. Behavioral analysis of the effects of group and emotional distractor valence on Stroop [incongruent-congruent] accuracy and RT were evaluated in a 2 (Group: YMP, control) × 2 (Valence: negative, neutral) ANOVA.

### fMRI methods

A 3T General Electric Signa EXCITE HD scanner (Milwaukee, WI) equipped with 40mT/m gradients was used for image acquisition. At the start of each fMRI session, a high-resolution three-dimensional fast spoiled gradient recalled echo (3D-FSPGR) anatomical sequence was collected (FOV = 25.6 cm, matrix = 256^2^, flip angle = 12°, 166 slices, slice thickness = 1 mm). BOLD functional images were collected for 34 contiguous slices parallel to the horizontal plane connecting the anterior and posterior commissures. A gradient-recalled inward spiral pulse imaging sequence was used (34 slices, TR = 1500 ms, TE = 30 ms, FOV = 25.6 cm, matrix = 64 × 64, flip angle = 60°, slice thickness = 3.8 mm, resulting in 4 × 4 × 3.8mm voxels).

Preprocessing was conducted using statistical parametric mapping software (SPM8; Wellcome Department of Imaging Neuroscience, London) to attenuate noise and artifacts. The first four volumes of each run were discarded to allow for T1 stabilization. All functional images underwent correction for acquisition timing and for head motion using rigid-body rotation and translation (Friston et al., 1994). Each participant's data was then subsequently warped into a standard stereotaxic space (Montreal Neurological Institute) with an isotropic 2 mm voxel size and smoothed with an 8 mm FWHM Gaussian filter.

### fMRI data analysis

Participant's data from each session was entered into a first-level, whole-brain analysis using the General Linear Model (Friston et al., 1994) to examine BOLD response to each of the six trial types; negative view, neutral view, negative congruent, neutral congruent, negative incongruent, neutral incongruent. For the first-level model, each event of each trial was modeled as a delta regressor at the onset of the event and convolved with a canonical hemodynamic response function. Motion was removed through rigid body rotation and translation and included as covariates, and a high-pass filter (128 s; 0.008 Hz) was applied to remove slow signal drift. Statistical images were thresholded with a mask containing regions of interest (ROI) that have been previously found to play a role in emotion-cognition interactions (Drevets, 2000; Ochsner et al., 2002, 2004; Yamasaki et al., 2002; Dolcos and McCarthy, 2006; Wang et al., 2008). These included bilateral posterior, dorsal and paracingulate cortices; inferior, middle and superior frontal gyri; inferior parietal lobule (IPL); insula and amygdala. These ROI's were obtained from automated anatomical labeling (AAL) (Tzourio-Mazoyer et al., 2002) in Marina (Walter et al., 2003). The goal of the analyses were to identify effects of YM experience (YMP, controls) on (1) emotional information processing [e.g., viewing emotional images (negative, neutral)] and (2) emotional distraction on Stroop-BOLD response.

#### Analysis of group, emotional distraction and stroop effect

To examine the effects of group and emotional distraction on the neurocorrelates of the Stroop effect, a Stroop contrast image (incongruent-congruent) of the second numerical grid in the trial (decision making event) was created separately for (1) negative and (2) neutral emotional distractors trials at the first level—resulting in two contrast images; negative emotional and neutral emotional distractor Stroop contrast maps. Regressors for each event were entered into a 2 (Group: YMP, control) × 2 (Valence: negative, neutral) random effects ANOVA. Main effects of Group and Valence; and Group × Valence interactions were evaluated.

#### Analyses of group and emotion reactivity

To examine between group differences in brain activity while viewing negative emotional images, regressors for each event of interest (1st presentation of an image during a trial; negative, neutral) were entered into a 2 (Group: YMP, control) × 2 (Valence: negative, neutral) random effects ANOVA. Main effects of Group and Valence; and Group × Valence interactions were evaluated.

Results were thresholded using the total number of voxels from the complete set of ROI's (i.e., one ROI mask containing all regions indicated in the Materials and Methods). In all analyses, voxels were considered significant if they passed a statistical threshold of *p* < 0.05 cluster-corrected. Cluster size for the comparisons was determined using AlphaSim and running 1000 Monte Carlo simulations (Ward, 2000) (*p* < 0.005, uncorrected; 432-μL cluster of contiguous significant voxels).

#### Exploratory analysis of affect and BOLD response

To examine the relationship between change in affect during performance of the Affective Stroop task and BOLD response during negative emotional viewing trials, a zero-order correlation was computed between % BOLD signal change in the amygdala cluster identified in the main effects model and the change score in positive affect (pre-post task self-report). We further explored this association using a multiple regression model to test whether the relationship between change in affect and BOLD response during negative emotional viewing was moderated by meditation experience. We regressed change in positive affect on the following set of variables: % signal change (BOLD response) to negative emotional viewing (the independent variable), a dichotomous variable coded 1 for controls and 2 for YMP (the moderator), and a group membership X BOLD response to negative emotional viewing interaction term. The significance of the interaction term indicated the presence of a moderation effect which was then explored graphically by plotting the regression lines (Baron and Kenny, 1986).

## Results

### Study participants

YMP and CG participants did not significantly differ with regard to demographics or measures of trait and state affect (see Table 1). Among the YMP group, age was not significantly correlated with years of meditation practice (*r* = −0.01, *p* = 0.98) or yoga (*r* = −0.27, *p* = 0.55).

**Table 1:** **Subject demographics and self report**.

	**Yogis (*n* = 7)**	**Controls (*n* = 7)**	
# Female	6	6	
Mean Age (SD)	36.4 (11.9)	35.5 (7.1)	
Years of Education (SD)	15.5 (2.5)	15.3 (2.3)	
Years of Yoga (SD)	9.3 (2.4)	0	
Years of Meditation (SD)	5.6 (4.2)	0	
**BASELINE MOOD**			
BAI	14.4 (2.5)	12.5 (1.9)	*ns* [>0.15]
CESD	3.4 (3.8)	2.6 (3.2)	*ns* [>0.6]
MAAS	4.9 (0.3)	5.0 (0.4)	*ns* [>0.5]
PANAS: Positive	35.6 (9.0)	36.1 (10.3)	*ns* [>0.9]
PANAS: Negative	10.4 (0.8)	10.7 (1.9)	*ns* [>0.7]
**STATE MOOD**			
PANAS: Positive	33.6 (10.8)	36.4 (7.6)	*ns* [>0.6]
PANAS: Negative	10.4 (0.8)	10.3 (0.5)	*ns* [>0.7]

### Behavioral data

No significant differences in Stroop RT were observed: there was no main effect of group [YMP (11.65ms), and controls (14.3ms)] or valence [negative distractor (15.7ms) and neutral distractor (10.3 ms)], nor was there a significant group X valence interaction on RT (all *p*-values > 0.1). Similarly, no significant differences in Stroop Accuracy were observed: there was no main effect of group [YMP (0.002), and controls (0.04)] or valence [negative distractor (0.02) and neutral distractor (0.02)], nor was there a significant or group X valence interaction on accuracy (all *p*-values > 0.2) (Table 2).

**Table 2:** **Affective Stroop task: behavioral stroop performance (incongruent-congruent)**.

**Negative**		**Neutral**		**Valence × Group Interaction**		**Main effect of Group**				**Main effect of Valence**			
**Yogis**	**Controls**	**Yogis**	**Controls**	**F**	***p***	**Yogis**	**Controls**	**F**	***p***	**Negative**	**Neutral**	**F**	***p***
**REACTION TIMES**													
30.8 (108)	0.7 (63)	−7.5 (56)	28 (54)	2.2	0.16	11.6	14.3	0.007	0.9	15.7	10.3	0.06	0.8
**ACCURACY**													
0.03 (0.11)	0.01 (0.07)	0.04 (0.1)	0.00 (0.08)	0.14	0.7	0.002	0.04	1.8	0.2	0.02	0.02	0.02	0.8

### Analysis of BOLD response during viewing trials

#### Interaction results

**Figure 2:**
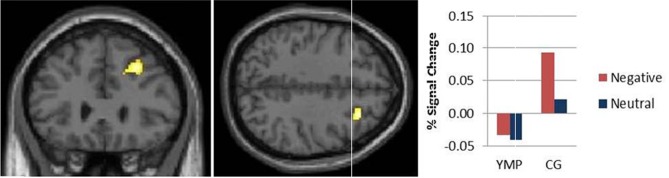
**fMRI contrast of the Group × Valence interaction on BOLD response during viewing emotional images.** Among the control group, activation was greater during negative versus neutral images and as compared to YMP in right middle frontal gyrus (MFG) (*x* = 28, *y* = 28, *z* = 40).

BOLD response to distractor images was modulated by a group X distractor valence interaction in right dlPFC [i.e., middle frontal gyrus] (effect size: *d* = 2.09) [see Table 3; Figure 2]: the CG were found to have greater activation to negative as compared to neutral emotional images, whereas the YMP group exhibited comparatively decreased activation to both negative and neutral emotional images.

**Table 3:** **BOLD response to viewing trials**.

**Contrast**	**Side**	**Lobe**	**Brain Region**	**Brodmann Area**	**MNI (*x*, *y*, *z*)**	**Cluster Size (mm^3^)**	***Z* (max)**	**Effect Size (d)**
**GROUP × VALENCE INTERACTION**								
	R	Frontal	dlPFC (MFG)	8	28 28 40	840	3.53	2.09
**MAIN EFFECT OF GROUP**								
none								
**MAIN EFFECT OF VALENCE**								
Neg > Neut	L	Limbic	Hippocampus		−30 2 −16	648	4.1	
			Amygdala		−24 −4 −14			
	R	Limbic	Insula (posterior)	13	38 −16 14	552	4.11	
	R	Frontal	Insula (anterior)		44 14 −10	1272	3.75	
Neut > Neg	none							

#### Main effects

**Figure A1:**
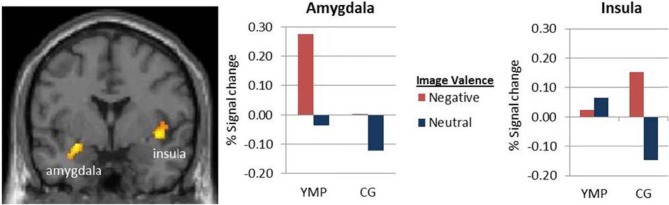
**fMRI contrast of the main effect of valence on viewing emotional images.** BOLD response was greater during negative, as compared to neutral emotional images in left Amygdala [*x* = −24, *y* = −4, *z* = −14] and right Insula [*x* = 38, *y* = −16, *z* = 14] *p* < 0.05 (cluster corrected).

A significant main effect of distractor valence was identified in left hippocampus and amygdala and right insula, characterized by greater activation to negative as compared to neutral images [see Table 3; Figure A1]. No main effect of group was found.

#### Exploratory correlational analyses

Exploratory analyses were performed to evaluate correlations between % signal change in dlPFC and amygdala clusters identified in the prior analyses during viewing trials. Across YMP and CG participants, a significant negative correlation was found between % signal change in the dlPFC and amygdala during negative (*r* = −0.575, *p.032-tailed*) but not neutral (*r* = −0.05, *p*.8) distractor images.

### Analysis of the effects of emotional distraction on stroop-BOLD response

#### Interaction results

**Figure 3:**
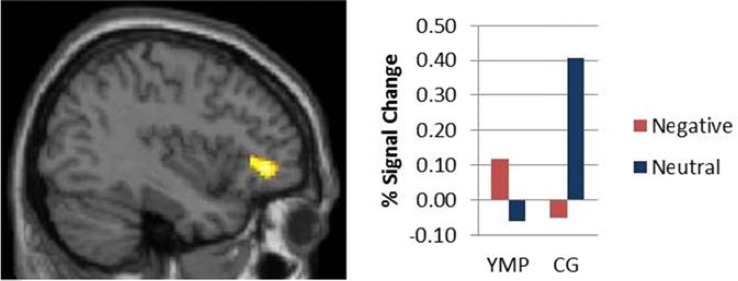
**fMRI contrast of the Group × Distractor Valence interaction on Stroop-BOLD response.** Among Yoga Meditation Practitioners (YMP), Stroop-BOLD was greater during negative, as compared to neutral Stroop-BOLD was greater during negative, as compared to neutral distractor trials in left Inferior Frontal Gyrus (IFG) [*x* = −38, *y* = 40, *z* = −2], whereas the opposite pattern was observed among the Control Group (CG).

Stroop-BOLD response was modulated by a group X distractor valence interaction in left ventrolateral prefrontal cortex (vlPFC) [i.e., inferior frontal gyrus] (effect size: *d* = 2.4) [see Table 4; Figure 3]: YMP exhibited greater Stroop-BOLD when negative, as compared to neutral emotional distractor images were presented, whereas the CG had greater Stroop-BOLD response during trials that presented neutral as compared to negative emotional distractors.

**Table 4:** **Stroop-BOLD response**.

**Contrast**	**Side**	**Lobe**	**Brain Region**	**Brodmann Area**	**MNI (*x*, *y*, *z*)**	**Cluster Size (mm^3^)**	***Z* (max)**	**Effect Size (d)**
t**STROOP EFFECT:GROUP × VALENCE INTERACTION**								
	L	Frontal	vlPFC (IFG)	10	−38 40 −2	824	3.67	2.45
**Main EFFECT OF GROUP**								
CG > YMP	L	Frontal	Superior Frontal Gyrus	10	−12 60 20	960	3.44	
YMP > CG	none							
**MAIN EFFECT OF VALENCE**								
Neg > Neut	L	Frontal	vlPFC (IFG)	9	−38 6 26	2488	3.94	
	L	Frontal	Anterior Cingulate	32	−8 12 34	856	3.63	
	R	Frontal	Anterior Cingulate	32	12 20 32	656	3.17	
Neut > Neg	none							

#### Main effects

A significant main effect of group was identified in left superior frontal gyrus (SFG), such that the CG had greater Stroop-BOLD response as compared to the YMP group [see Table 4]. A significant main effect of distractor valence on Stroop-BOLD response was identified in left vlPFC and bilateral anterior cingulate cortex (ACC), such that negative distractor Stroop trials elicited greater activation than neutral distractor Stroop trials [see Table 4].

### Exploratory analysis of self-reported affect and emotional view trial BOLD response

**Figure 4:**
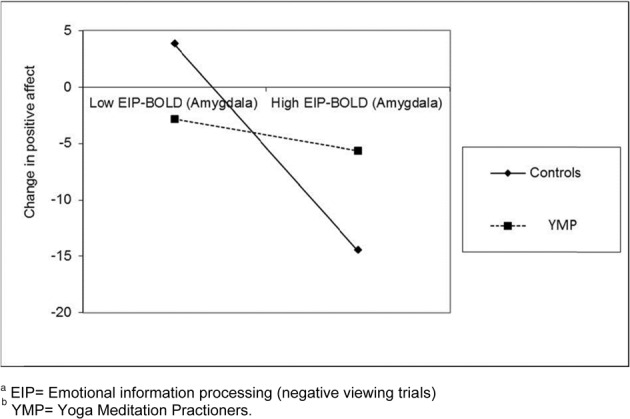
**Plot of interaction between meditation experience and BOLD response in the amygdala to viewing negative emotional images on change in positive affect during participation in the Affective Stroop task^ab^**.

No significant main effects of time or group X time interaction effects were observed for change in negative affect from baseline through completion of the Affective Stroop task. In contrast, a significant main effect of time on positive affect was observed; across YMP and controls, positive affect decreased significantly from baseline through completion of the Affective Stroop task, [*F*_(1, 12)_ = 5.54, *p* = 0.04]. The group X time interaction was nonsignificant, [*F*_(1, 12)_ = 0.78, *p* = 0.40]. Across the entire sample, change in positive affect from baseline through completion of the Affective Stroop task was significantly correlated with BOLD response to negative distractors in the left amygdala, *r* = 0. 58, *p* = 0.03 (Figure 4). Yet, in exploratory moderation analyses, a significant group X emotional view-BOLD interaction on change in positive affect was observed, *B* = −3.31, *SE* = 1.17, *p* = 0.018 (see Figure 3). Graphical inspection of the plot of the interaction effect and *post-hoc* probing of the simple slopes (Aiken and West, 1991) indicated that among controls, higher BOLD response to viewing negative emotional images was significantly related to larger decreases in positive affect during participation in the Affective Stroop task (*t* = 3.75, *p* = 0.004). Simple slopes analysis revealed no such significant relationship between BOLD response to viewing negative emotional images and affective reactivity among YMP (*t* = 1.13, *p* = 0.29).

## Discussion

The present study represents one of the first attempts to discriminate YMP from meditation-naïve subjects on the basis of the neural substrates of negative emotional reactivity and emotion-cognition interactions. Though the study failed to identify any significant task-related behavioral findings, it did identify a number of significant task-related neural differences between groups—suggesting within the context of this study that the groups differed from one another, not on *how well* they performed the task, but rather on *how* they performed the task. The study yielded three main findings. First, YMP were less reactive in right dlPFC (i.e., MFG) during viewing negative emotional images. Secondly, during a cognitively demanding task, the presence of emotionally irrelevant distractor images resulted in greater vlPFC (i.e., IFG) activation in YMP. Thirdly, among meditators amygdala activation to negative emotional distracters was uncoupled with task-related changes in affect, unlike non-meditators whose decreases in positive affect were correlated with increased amygdala activation. Taken together, these data suggest that YMP may selectively recruit dissociable frontal executive-dependent strategies in response to emotionally salient information as a function of cognitive demands and not during emotional processing *per se*. Furthermore, bottom-up driven emotional responding among YMP does not have downstream effects on later mood state, dissimilar to that observed among controls.

### Selective recruitment of frontal executive mechanisms

#### Emotional information processing

In the present study, YMP exhibited less activation in right dlPFC (i.e., MFG) in response to all distractors images, whereas controls had heightened activation to negative emotional distractors. The MFG is involved in attention (Cabeza and Nyberg, 2000), cognitive control (Kerns et al., 2004), goal directed processes (Blair et al., 2007) and exerting cognitive control over emotional processes (e.g., emotion regulation) (Ochsner et al., 2002; Ochsner and Gross, 2008). With regard to emotion regulation, the mediation hypothesis posits that executive circuitry (e.g., dlPFC) reduces negative affective response by top-down modulation of affective circuitry (Wager et al., 2008), marked by activation in dlPFC that is inversely related to activation in the amygdala. The current study finding that % signal change in dlPFC was negatively correlated with % signal change in the amygdala during negative, but not neutral, image trials is consistent with the meditation hypothesis model. Moreover, though controls exhibit a reciprocal pattern of executive-limbic BOLD response during viewing negative emotional images, YMP do not. Though the experimental task was not designed to probe *how* subjects processed the emotional images, this pattern of brain activation is consistent with the notion that mindfulness, whether generated via yoga postures or sitting meditation, increases attention toward emotion without active attempts to cognitively restructure affective experience (Hölzel et al., 2011). Recent neuroimaging evidence suggests that mindfulness practitioners evidence decreased fronto-executive activation during processing of emotionally aversive experiences (e.g., pain) (Gard et al., 2011). Such attention to emotional information without cognitive control may reflect the attitude of acceptance and nonjudgment that is held to be an essential component of yoga and mindfulness.

#### Emotion-cognition interactions

With regard to the effects of negative emotion processing on cognition, YMP had greater Stroop-Bold response in left vlPFC (i.e., IFG) when negative, as compared to neutral, emotional distractors were presented; whereas controls exhibited the opposite pattern. The vlPFC (i.e., IFG) is part of a network involved in inhibitory control (Aron and Poldrack, 2006; Aron et al., 2007), social emotional processes (Carr et al., 2003), and cognitive control over emotional distraction (Dolcos and McCarthy, 2006). Plausibly, the observed pattern of brain activation may indicate that YMP selectively recruited neurocognitive resources to disengage from negative emotional information processing and engage the cognitive demands presented by the Stroop task. Increased activity in the vlPFC may prevent working memory functions from becoming disturbed by incoming sensory input stemming from negative emotional stimuli by deactivating emotional information processing signals ascending from subcortical routes to the amygdala (Austin, 2009). In contrast, CG participants marshaled comparatively fewer frontal-executive resources to resolve emotional interference in the face of this demanding task.

In conjunction, these findings suggest a brain model associated with YM practice whereby frontal executive-dependent strategies to reduce emotional processing are selectively implemented as a function of whether competing cognitive demands are presented. In other words, in the absence of concurrent task performance, YMP appear to process emotional information without effortful cognitive control; however, when emotional experience occurs within the context of a demanding task situation, YMP may resolve emotional interference via recruitment of regions of cortex that subserve cognitive control. Plausibly, this strategy would ensure neurocognitive resource efficiency and confer significant behavioral advantages, such as the psychological benefits observed in clinical and non-clinical samples (Chiesa and Serretti, 2010).

### Impact of limbic response on subsequent affect

Exploratory analyses revealed that Affective Stroop performance was associated with degradation of positive affect over time and non-significant effects on negative affect. This decrease in positive state affect was likely the result of exposure to aversive images coupled with engagement in a cognitively demanding task. These findings are consistent with prior literature on emotion-cognition interactions (Holdwick and Wingenfeld, 1999; Calkins et al., 2011). For example, Calkins et al. (2011) found that individuals participating in a cognitive control task experienced a significant decrease in positive affect after a negative mood induction, but reported a trend toward a lower induction of negative mood following mood induction. Hence, cognitive control tasks may operate as a buffer from subsequent negative mood induction, by virtue of the fact that they engage regions of prefrontal cortex involved in downregulation of negative emotion. Such prefrontal control could protect individuals from negative emotional reactivity in the context of a cognitive and affective challenge. Alternatively, lack of significant task-related changes in negative affect may stem from low statistical power driven by the modest sample size in this study.

During viewing negative emotional images, CG participants exhibited a stereotypic limbic-mediated affective response, such that increased activation in amygdala to negative emotional distractors predicted greater decay of positive affect over the task session. In contradistinction, YMP amygdala responses during viewing negative emotional images were uncoupled with changes in positive affect. Conceptually, this finding complements the lack of dlPFC activation observed among YMP during exposure to negative emotional distractors. If YMP can process emotional information without effortful cognitive control through mindful awareness and acceptance of experience, they may avoid the negative consequences of response-focused forms of emotion regulation like suppression (Wenzlaff and Wegner, 2000; Gross, 2002; Campbell-Sills et al., 2006), which has been shown to deplete neurocognitive resources during affective cue-exposure (Garland and Roberts-Lewis, 2012).

### Conclusion and limitations

The present study included a well-controlled, matched sample of YMP and YM naive subjects and a neuroimaging paradigm that allows for modeling of the interactive effects of emotion on cognition—an area of research currently underrepresented in the literature.

However, limitations included a relatively small sample size and the use of a cognitive paradigm with sufficient task difficulty to subjects which may have attenuated the ability to detect behavioral Stroop effects. The small sample may have limited the reliability of study findings on the observed impact of limbic responses on subsequent affect, and thus this analysis should be replicated in studies with larger sample sizes. Moreover, the cross-sectional design of the current study cannot elucidate whether the observed group differences in neurocognitive function reflect trait-level factors linked with the initiation and maintenance of long-term YM practice, or whether these differences are the result of recurrent yoga practice over time. However, if these findings reflect differences that are a result of recurrent practice over time, they suggest that yoga mediation practice may provide putative therapeutic benefits for individuals with dysregulated affect and/or cognitive control deficits. One such example may be individuals with a substance abuse disorder. For example, the extant research on the neurobiology of substance abuse disorders posits that chronic drug use is associated with dysregulated prefrontal-dependent cognitive control function, which may play a key role in negative affect and inhibitory control (Koob and Volkow, 2010). To test these hypotheses, large-scale longitudinal studies are needed to follow individuals as they initiate the discipline of YM practice and cultivate progressively deeper states of mindfulness over time.

### Conflict of interest statement

Drs. Froeliger, Garland and McClernon report having research funding from the National Institute on Drug Abuse. Ms. Modlin reports no conflicts of interest.
